# The effect of tofacitinib on pneumococcal and influenza vaccine responses in rheumatoid arthritis

**DOI:** 10.1136/annrheumdis-2014-207191

**Published:** 2015-03-20

**Authors:** Kevin L Winthrop, Joel Silverfield, Arthur Racewicz, Jeffrey Neal, Eun Bong Lee, Pawel Hrycaj, Juan Gomez-Reino, Koshika Soma, Charles Mebus, Bethanie Wilkinson, Jennifer Hodge, Haiyun Fan, Tao Wang, Clifton O Bingham

**Affiliations:** 1 Division of Infectious Diseases, Oregon Health and Science University, Portland, Oregon, USA; 2 Department of Internal Medicine and Rheumatology, Healthpoint Medical Group, Tampa, Florida, USA; 3 Department of Rheumatology, Osteo-Medic s.c, Bialystok, Poland; 4 Department of Rheumatology, Bluegrass Community Research Inc., Lexington, Kentucky, USA; 5 Department of Internal Medicine, Seoul National University, Seoul, South Korea; 6 Department of Rheumatology and Clinical Immunology, Poznan University of Medical Sciences, Poznan, Poland; 7 Rheumatology Department, Hospital Clinico Universitario, Santiago de Compostela, Spain; 8 Department of Clinical Sciences, Pfizer Inc, Groton, Connecticut, USA; 9 Department of Inflammation, Pfizer Inc, New York, New York, USA; 10 Pfizer Inc, Shanghai, China; 11 Division of Rheumatology, Department of Medicine, Johns Hopkins University, Baltimore, Maryland, USA

**Keywords:** Rheumatoid Arthritis, Vaccination, Infections

## Abstract

**Objective:**

To evaluate tofacitinib's effect upon pneumococcal and influenza vaccine immunogenicity.

**Methods:**

We conducted two studies in patients with rheumatoid arthritis using the 23-valent pneumococcal polysaccharide vaccine (PPSV-23) and the 2011–2012 trivalent influenza vaccine. In study A, tofacitinib-naive patients were randomised to tofacitinib 10 mg twice daily or placebo, stratified by background methotrexate and vaccinated 4 weeks later. In study B, patients already receiving tofacitinib 10 mg twice daily (with or without methotrexate) were randomised into two groups: those continuing (‘continuous’) or interrupting (‘withdrawn’) tofacitinib for 2 weeks, and then vaccinated 1 week after randomisation. In both studies, titres were measured 35 days after vaccination. Primary endpoints were the proportion of patients achieving a satisfactory response to pneumococcus (twofold or more titre increase against six or more of 12 pneumococcal serotypes) and influenza (fourfold or more titre increase against two or more of three influenza antigens).

**Results:**

In study A (N=200), fewer tofacitinib patients (45.1%) developed satisfactory pneumococcal responses versus placebo (68.4%), and pneumococcal titres were lower with tofacitinib (particularly with methotrexate). Similar proportions of tofacitinib-treated and placebo-treated patients developed satisfactory influenza responses (56.9% and 62.2%, respectively), although fewer tofacitinib patients (76.5%) developed protective influenza titres (≥1:40 in two or more of three antigens) versus placebo (91.8%). In study B (N=183), similar proportions of continuous and withdrawn patients had satisfactory responses to PPSV-23 (75.0% and 84.6%, respectively) and influenza (66.3% and 63.7%, respectively).

**Conclusions:**

Among patients starting tofacitinib, diminished responsiveness to PPSV-23, but not influenza, was observed, particularly in those taking concomitant methotrexate. Among existing tofacitinib users, temporary drug discontinuation had limited effect upon influenza or PPSV-23 vaccine responses.

**Trial registration numbers:**

NCT01359150, NCT00413699.

## Introduction

Patients with rheumatoid arthritis (RA) are at increased risk of serious infection due to their underlying disease, and in some cases, their immunomodulatory therapies.^1–10^ Recommendations exist to vaccinate patients with RA against pneumococcus and influenza, and it is common for studies to evaluate the immune responses to these vaccines in the context of disease-modifying antirheumatic drug (DMARD) therapy.^11–13^ Such studies provide relevant information regarding vaccine timing for practicing clinicians.

Tofacitinib is an oral Janus kinase (JAK) inhibitor for the treatment of RA.^14–19^ To investigate its effect on vaccine response and to provide effective clinical guidance regarding the timing of vaccinations in conjunction with tofacitinib use, two studies involving pneumococcal polysaccharide and influenza vaccines were conducted to address the following questions in common clinical scenarios: (A) For tofacitinib-naive patients with RA, are vaccine responses diminished by tofacitinib such that patients should be vaccinated prior to drug start? (B) For patients with RA receiving tofacitinib, is it necessary to stop therapy while giving vaccinations?

## Methods

### Study designs

We conducted two independent studies evaluating vaccine responses in relation to tofacitinib use. Study A (A3921129; NCT01359150) was a randomised, double-blind, placebo-controlled, phase II study. Study B (A3921024; NCT00413699) was a vaccine substudy of an ongoing, open-label, multicentre, long-term extension (LTE) study (study ongoing; database unlocked) that included patients who had participated in a prior qualifying index study of tofacitinib. In both studies, all patients met the 1987 American College of Rheumatology criteria for RA.^20^ Patients with influenza vaccination in the last 6 months or pneumococcal vaccination in the last 5 years were excluded.

**Figure 1:**
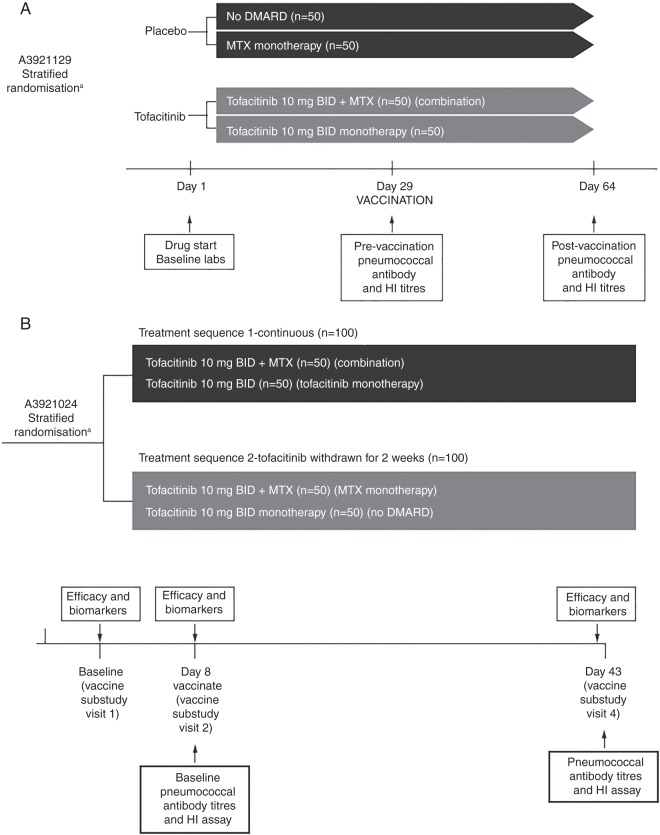
(A) Study A (tofacitinib-naive patients) design evaluating vaccine responses in patients randomised to tofacitinib vs placebo. (B) Study B (patients using tofacitinib) randomisation schemata. Patients were randomised to one of two groups: the ‘continuous’ group, which received tofacitinib without interruption, or the ‘withdrawn’ group, in which tofacitinib was withdrawn for 1 week at randomisation and then resumed 1 week after vaccination. ^a^Stratified by background methotrexate (MTX) use. BID, twice daily; DMARD, disease-modifying antirheumatic drug; HI, haemagglutination inhibition.

Study A was conducted among tofacitinib-naive patients with RA. Patients were randomised 1:1 to receive either tofacitinib 10 mg twice daily or placebo and stratified according to current background methotrexate use (figure 1A). Baseline serum pneumococcal and influenza antibody titres were collected 4 weeks after drug start (ie, day 29 after tofacitinib or placebo start), after which patients were vaccinated with influenza vaccine and the 23-serotype pneumococcal polysaccharide vaccine (PPSV-23). Influenza and pneumococcal antibody titres were again collected 35 days after vaccination (day 64 after drug start; figure 1A).

Study B was conducted among a subgroup of patients with RA participating in an ongoing LTE tofacitinib trial. Patients received tofacitinib 10 mg twice daily for ≥3 months prior to entry into this vaccination substudy (median tofacitinib treatment duration at study entry was approximately 22–23 months). Patients were stratified by background methotrexate use and randomised 1:1 to one of two groups: the ‘continuous’ group, which received tofacitinib without interruption, or the ‘withdrawn’ group, in which tofacitinib was withdrawn at the time of randomisation for 1 week prior to receiving the influenza and pneumococcal vaccinations, and then resumed 1 week after vaccination (figure 1B). Baseline serum pneumococcal and influenza antibody titres were collected 1 week after randomisation (day 8), immediately after which patients were vaccinated with influenza vaccine and PPSV-23. Influenza and pneumococcal antibody titres were collected again 35 days after vaccination (figure 1B).

For both studies, background methotrexate use was defined as continuous (>4 months) and stable dosage (≥10 mg/week and ≤25 mg/week for ≥6 weeks) prior to the first dose of study drug; prednisone (<10 mg/day) was allowed; neither methotrexate nor prednisone dose adjustments were permitted during the studies.

### Vaccines and immunogenicity endpoints

For each study, PPSV-23 (Pneumovax, Merck & Co Inc, Whitehouse Station, New Jersey, USA) and the 2011–2012 seasonal trivalent-inactivated influenza vaccine for the Northern hemisphere (A/California/7/2009 (H1N1), A/Perth/16/2009 (H3N2), B/Brisbane/60/2008; Fluzone, Sanofi Pasteur, Lyon, France) were used. Measurement of influenza haemagglutination inhibition (HI) and antipneumococcal antibody titres (ELISA for anti-pneumococcal capsular polysaccharide (anti-PSS) immunoglobulin G (IgG) antibodies) was performed by the Focus Diagnostic (Cypress, California, USA) and Pfizer Vaccine Research Labs (Pearl River, New York, USA), respectively.

In both studies, the same immunogenicity outcome measures were evaluated at the vaccination and post-vaccination visits, and the same definitions of satisfactory vaccine responses were used.

The primary outcome for each vaccine was the proportion of patients achieving a satisfactory humoral response 35 days after vaccination. ‘Satisfactory response’ was *a priori* defined for pneumococcal vaccine as a twofold or more increase from vaccination baseline in antibody concentrations in six or more of 12 pneumococcal serotypes (1, 3, 4, 5, 6B, 7F, 9V, 14, 19A, 19F, 23F and 18C), and for influenza vaccine as a fourfold or more increase in HI antibody titres in two or more of three influenza antigens (A/H1N1, A/H3N2, B).

Secondary endpoints included: the proportion of patients who developed protective HI titres to influenza (‘seroprotection’ defined as ≥1:40 influenza antibody titre in two or more of three antigens^21–23^) and the post-vaccination geometric mean fold rise (GMFR) in antibody titres.

### Statistical analyses

In both studies, immunogenicity analyses were performed among the evaluable population. The evaluable population were those patients who were randomised, received vaccination at baseline, and for whom antibody assay results both before and after vaccination as per protocol were obtained. For primary outcome measures, the percentages of patients having satisfactory responses at 35 days after vaccination were summarised for each treatment group. To evaluate the treatment effect between groups, the point estimate for the treatment difference and the associated exact 95% CIs, computed using the unconditional exact method were also provided.^24^ The same methodology was used to evaluate binary secondary outcomes, including the presence of protective HI titres. For the secondary outcome of GMFR, which was calculated by the geometric mean titre from pre- to post-vaccination time points to each pneumococcal serotype and influenza antigen, the geometric mean and associated 95% CI (from the back transformation of the CI at the logarithmic scale) for the fold rise were presented for each treatment group and for each antigen or serotype. A subgroup analysis by background methotrexate use was also performed for the primary and secondary outcomes. All analyses were conducted using SAS version 9.2 (SAS Institute Inc, Cary, North Carolina, USA).

### Treatment groups and exposure subgroup definitions

All outcomes were evaluated according to treatment group in study A (tofacitinib vs placebo) and in study B (continuous vs withdrawn), and according to the subgroups of background methotrexate use. Therefore, both studies functionally contained four similar exposure subgroups, herein referred to as follows: (1) no DMARD (ie, lacking both methotrexate and tofacitinib), (2) methotrexate monotherapy, (3) tofacitinib monotherapy and (4) combination tofacitinib/methotrexate therapy.

## Results

### Study A: patients naive to tofacitinib

A total of 223 patients were enrolled into study A and 200 patients (tofacitinib n=102, placebo n=98) were included in the evaluable population. Demographic and baseline characteristics of evaluable patients randomised to tofacitinib 10 mg twice daily or placebo were similar, with the exception of a greater proportion of placebo-treated patients having evidence of pre-existing seroprotection to influenza (table 1).

**Table 1:** Demography and baseline characteristics of evaluable patients in study A (patients naive to tofacitinib) and study B (patients using tofacitinib)

	Study A		Study B	
			Tofacitinib 10 mg twice daily	
	Tofacitinib 10 mg twice daily (N=102)	Placebo (N=98)	Continuous (N=92)	Withdrawn (N=91)
Female, n (%)	75 (73.5)	79 (80.6)	78 (84.8)	79 (86.8)
Age in years, median (range)	53 (25–82)	53 (23–77)	57.0 (28–78)	54.0 (24–72)
DAS28-4 (ESR), mean (SD)	6.03 (1.05)	5.78 (1.10)	3.64 (1.36)	3.71 (1.34)
Background MTX, n (%)	57 (55.9)	55 (56.1)	55 (59.8)	55 (60.4)
Prednisone use, n (%)	38 (37.3)	31 (31.6)	39 (42.4)	46 (50.5)
Evidence of influenza seroprotection,* n (%)	20 (19.6)	32 (32.7)	22 (23.9)	23 (25.3)

*Protection status (1:40 in two or more of three antigens) to influenza vaccine at 35-days after vaccination.

DAS, disease activity score; ESR, erythrocyte sedimentation rate; MTX, methotrexate.

#### Pneumococcal responses

**Figure 2:**
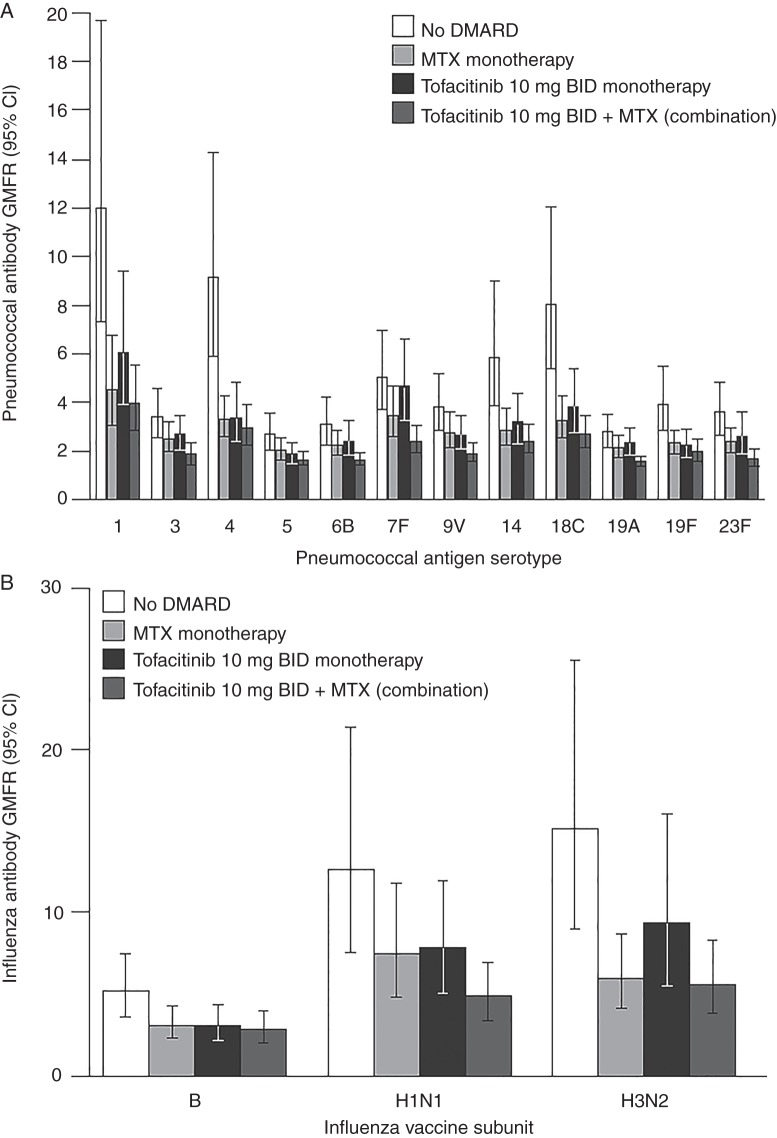
(A) Study A (tofacitinib-naive patients) pneumococcal serotype-specific titre geometric mean fold rise (GMFR) from vaccination baseline and 95% CI at 35 days after vaccination, by exposure subgroup. (B) Study A (tofacitinib-naive patients) influenza antibody (haemagglutination inhibition) titre GMFR and 95% CI 35 days after vaccination, by exposure subgroup. BID, twice daily; DMARD, disease-modifying antirheumatic drug; GMFR, geometric mean fold rise; MTX, methotrexate.

Overall, pneumococcal vaccination responses were reduced for tofacitinib-treated patients, whereby 46 patients (45.1%) developed a satisfactory response compared with 67 (68.4%) placebo-treated patients (−23.3% difference (95% CI −36.6% to −9.6%)). Among exposure subgroups, the highest responses were observed among the no DMARD group, followed by reductions of similar magnitude in the tofacitinib and methotrexate monotherapy subgroups. The greatest reduction was observed in combination tofacitinib/methotrexate patients for whom only 18 (31.6%) patients developed satisfactory responses (table 2). For pneumococcal GMFR responses, similar trends were noted (figure 2A).

**Table 2:** Study A (patients naive to tofacitinib) primary endpoint: proportion of patients achieving satisfactory* humoral response to pneumococcal and influenza vaccines at 35 days after vaccination, by treatment group and stratified by background MTX use

	Tofacitinib 10 mg twice daily (N=102)	Placebo (N=98)	Percentage difference between treatment groups (95% CI)
PPSV-23 vaccine			
Overall, n (%)	46 (45.1)	67 (68.4)	−23.3 (−36.6 to −9.6)
Stratified by MTX use at baseline, n/N (%)			
Yes	18/57 (31.6)	34/55 (61.8)	−30.2 (−47.3 to −11.4)
No	28/45 (62.2)	33/43 (76.7)	−14.5 (−34.8 to 6.2)
Influenza vaccine			
Overall, n (%)	58 (56.9)	61 (62.2)	−5.4 (−19.3 to 8.5)
Stratified by MTX use at baseline, n/N (%)			
Yes	29/57 (50.9)	32/55 (58.2)	−7.3 (−25.9 to 11.4)
No	29/45 (64.4)	29/43 (67.4)	−3.0 (−24.0 to 17.4)

*Satisfactory response to pneumococcal vaccine defined as a twofold or more titre increase against six or more of 12 pneumococcal serotypes; satisfactory response to influenza vaccine defined as a fourfold or more titre increase against two or more of three influenza antigens.

MTX, methotrexate; PPSV-23, 23-valent pneumococcal polysaccharide vaccine.

#### Influenza responses

Overall, a similar proportion of patients receiving tofacitinib (56.9%) and placebo (62.2%) achieved satisfactory immune responses to influenza vaccination (–5.4% difference (95% CI −19.3% to 8.5%)). Similar proportions achieved satisfactory responses among exposure subgroups (table 2). The proportion achieving seroprotection at 35 days after vaccination was significantly higher in the placebo group (91.8%) than in the tofacitinib group (76.5%). Among exposure subgroups, the seroprotection rate was reduced only in the combination tofacitinib/methotrexate group; seroprotection rates by exposure subgroup were 90.7% (39/43 patients) for no DMARD, 92.7% (51/55 patients) for methotrexate monotherapy, 91.1% (41/45 patients) for tofacitinib monotherapy and 64.9% (37/57 patients) for combination tofacitinib/methotrexate.

When limiting this analysis to the subset of patients lacking seroprotection at baseline, 70.7% (58/82 patients) versus 87.9% (58/66 patients) in the tofacitinib and placebo groups, respectively, reached seroprotection. Among exposure subgroups that lacked baseline seroprotection, the proportions that developed seroprotection after vaccination were 86.7% (26/30 patients) for no DMARD, 88.9% (32/36 patients) for methotrexate monotherapy, 88.6% (31/35 patients) for tofacitinib monotherapy and 57.5% (27/47 patients) for combination tofacitinib/methotrexate.

When examining GMFR responses for each individual antigen, the lowest responses were consistently observed for influenza B antigen and were similar among exposure subgroups. Responses to the H1N1 and H3N2 vaccine components were more robust; patients using no DMARD had the highest responses, whereas lower and similar responses were observed in the tofacitinib and methotrexate monotherapy subgroups, as well as the combination tofacitinib/methotrexate subgroup (figure 2B).

### Study B: patients using tofacitinib

A total of 199 patients were enrolled into study B and 183 patients (tofacitinib continuous n=92, tofacitinib withdrawn n=91) completed the study and were evaluable. Continuous and withdrawn patients showed similar demographic and baseline characteristics at baseline (table 1).

#### Pneumococcal responses

**Figure 3:**
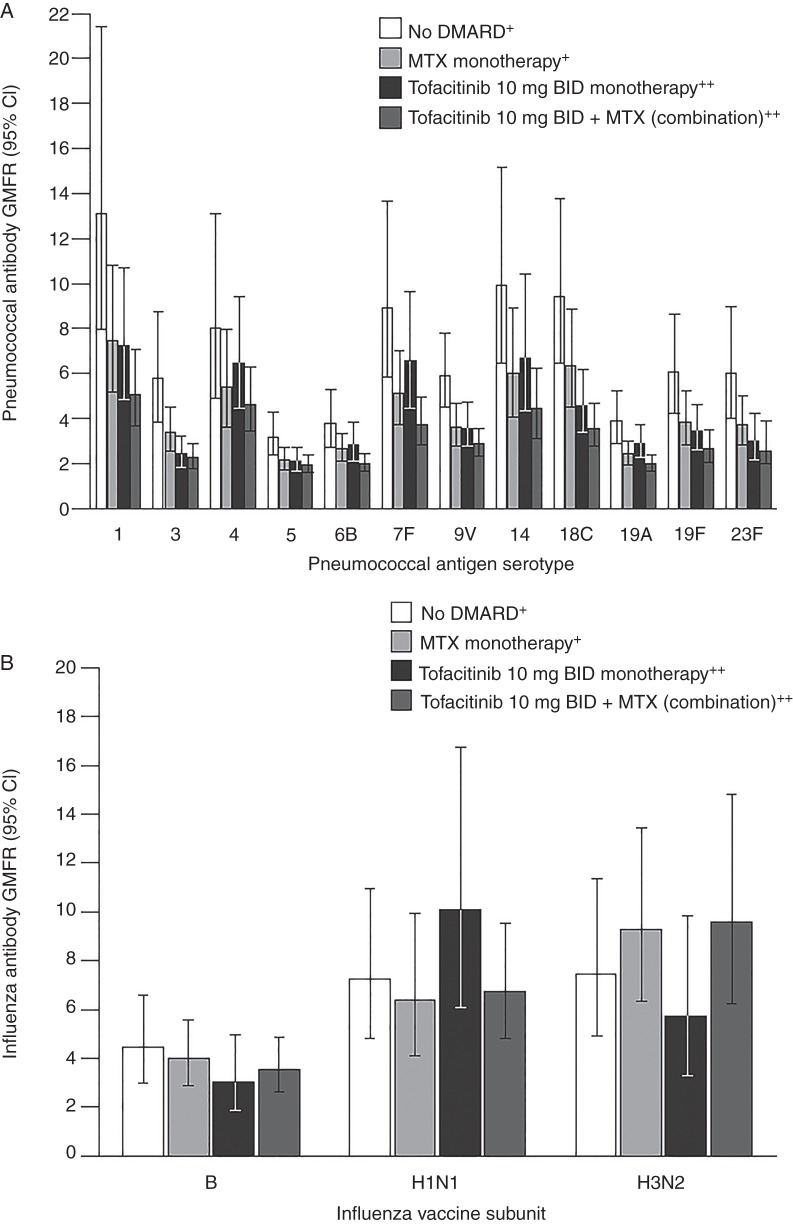
(A) Study B (patients using tofacitinib) pneumococcal serotype-specific titre geometric mean fold rise (GMFR) from vaccination baseline and 95% CI at 35 days after vaccination, by exposure subgroup. (B) Study B (patients using tofacitinib) influenza antibody (haemagglutination inhibition) titre GMFR and 95% CI 35 days after vaccination, by exposure subgroup. ^+^Tofacitinib withdrawn group; ^++^Tofacitinib continued group. BID, twice daily; DMARD, disease-modifying antirheumatic drug; GMFR, geometric mean fold rise; MTX, methotrexate.

Overall, the proportion of patients with satisfactory responses to pneumococcal vaccinations was reduced in the tofacitinib continuous group compared with the withdrawn group (75.0% vs 84.6%, respectively, −9.6% difference (95% CI −24.0% to 4.7%); table 3). As in study A, similar GMFR trends for each serotype were observed by exposure subgroup: the no DMARD subgroup had the highest GMFR responses, whereas the tofacitinib and methotrexate monotherapy subgroups had diminished and similar responses; the lowest responses were observed in the combination tofacitinib/methotrexate subgroup (figure 3A).

**Table 3:** Study B (patients using tofacitinib) primary endpoint: proportion of patients achieving satisfactory* humoral responses at 35 days after vaccination, by treatment subgroup (‘continuous’ and ‘withdrawn’) and stratified by background MTX use

	Tofacitinib 10 mg twice daily		Percentage difference between treatment groups (95% CI)
	Continuous (N=92)	Withdrawn (N=91)	
PPSV-23 vaccine			
Overall, n (%)	69 (75.0)	77 (84.6)	−9.6 (−24.0 to 4.7)
Stratified by MTX use at baseline, n/N (%)			
Yes	36/55 (65.5)	44/55 (80.0)	−14.5 (−33.3 to 5.0)
No	33/37 (89.2)	33/36 (91.7)	−2.5 (−25.2 to 20.0)
Influenza vaccine			
Overall, n (%)	61 (66.3)	58 (63.7)	2.6 (−12.2 to 16.6)
Stratified by MTX use at baseline, n/N (%)			
Yes	38/55 (69.1)	34/55 (61.8)	7.3 (−12.2 to 26.4)
No	23/37 (62.2)	24/36 (66.7)	−4.5 (−27.8 to 17.7)

*Satisfactory response to pneumococcal vaccine defined as a twofold or more titre increase against six or more of 12 pneumococcal serotypes; satisfactory response to influenza vaccine defined as a fourfold or more titre increase against two or more of three influenza antigens.

MTX, methotrexate; PPSV-23, 23-valent pneumococcal polysaccharide vaccine.

#### Influenza responses

Similar proportions of patients in the tofacitinib continuous and withdrawn groups achieved satisfactory immune responses to influenza vaccination at 35 days after vaccination (66.3% vs 63.7%, respectively, 2.6% difference (95% CI −12.2% to 16.6%); table 3). Evaluation by exposure subgroup revealed similar proportions of patients achieving a satisfactory response in the no DMARD (66.7%), tofacitinib monotherapy (62.2%), methotrexate monotherapy (61.8%) and combination tofacitinib/methotrexate (69.1%) subgroups (table 3).

Among secondary outcomes, 69 (75.0%) tofacitinib continuous and 75 (82.4%) withdrawn patients (−7.4% difference (95% CI −21.9% to 6.8%)) achieved seroprotection for influenza at 35 days after vaccination. When limiting this analysis to patients lacking baseline seroprotection, similar proportions of patients achieved this outcome in each treatment group with 47 (67.1%) and 52 (76.5%) continuous and withdrawn patients, respectively, achieving seroprotection. Similarly, among patients lacking baseline seroprotection, the proportion achieving seroprotection by exposure subgroup was 83.3% (20/24 patients) for no DMARD, 72.7% (32/44 patients) for methotrexate monotherapy, 60.7% (17/28 patients) for tofacitinib monotherapy and 71.4% (30/42 patients) for combination tofacitinib/methotrexate therapy.

Evaluation of GMFR responses by antigen also showed similar responses among each exposure subgroup (figure 3B).

## Discussion

We conducted two trials in patients with RA to evaluate the effect of tofacitinib on vaccine responses to PPSV-23 and the trivalent seasonal influenza vaccine. In tofacitinib-naive patients (study A), subsequent initiation of tofacitinib was associated with diminished responses to PPSV-23, particularly in patients taking concurrent methotrexate. For influenza vaccine responses, a decrease in response was noted only among tofacitinib-treated patients taking background methotrexate, although the proportion of patients achieving satisfactory immune responses was generally similar in all exposure subgroups. In study B, among current tofacitinib users, these data suggested that temporary discontinuation of tofacitinib for vaccination purposes had little impact on the immunogenicity of either vaccine, although small decreases in antipneumococcal titres were observed in patients with continued tofacitinib exposure, particularly those taking background methotrexate. Collectively, these studies suggest that tofacitinib diminished responses to PPSV-23, particularly when used in combination with methotrexate, and that tofacitinib treatment has limited effect on influenza vaccine responses.

These studies were conducted to address clinically relevant vaccination questions in two distinct patient groups: those in whom tofacitinib treatment is being considered and those currently receiving tofacitinib. With regard to PPSV-23 responses, similar trends were observed in both studies, although fewer patients achieved satisfactory PPSV-23 responses in tofacitinib-naive patients who started tofacitinib (study A). It is possible that the higher RA disease activity present in this study at the time of vaccination contributed to lower overall pneumococcal responses across all exposure subgroups in that study (table 1). Taken together, the two studies suggest that methotrexate and tofacitinib monotherapy result in similar reductions in responsiveness to the PPSV-23 vaccine, and when administered concomitantly, greater decreases in response occur. These trends were evident in both studies when looking at GMFR responses across exposure subgroups. For most serotypes, the greatest GMFR was reported in patients in the no DMARD subgroup, followed by lower and similar responses in the tofacitinib and methotrexate monotherapy subgroups, with the lowest GMFR reported in the combination tofacitinib/methotrexate subgroup.

However, it is important to note that in both studies, the majority of patients in all exposure subgroups reached the primary outcome measure and achieved satisfactory humoral responses to PPSV-23. The important exception was for patients in study A starting tofacitinib with background methotrexate: only 31.6% developed satisfactory responses to PPSV-23. Data from study A suggest that to maximise PPSV-23 responsiveness, clinicians should vaccinate prior to tofacitinib or methotrexate treatment, if possible. Data from study B, however, suggest relatively less diminishment in responses associated with tofacitinib such that temporary drug discontinuation in order to vaccinate would be of little benefit. It is possible that delaying tofacitinib resumption for a longer time period after vaccination could have promoted greater vaccine responses.

For influenza vaccination, similar proportions of patients in each study reached the primary outcome measure of satisfactory immune responses, irrespective of treatment group or exposure subgroup. Although in study A a decrease in response was noted for patients using tofacitinib and concomitant methotrexate, only 50.9% reached the primary endpoint compared with 67.4% in the no DMARD subgroup. Trends in GMFR decrease were also noted among the exposure subgroups using either drug (or both) compared with no DMARD. However, in study B, no consistent effects were noted in relation to treatment groups or exposure subgroups; both primary and secondary outcomes were unaffected by tofacitinib or methotrexate monotherapy or in combination. Taken collectively, these studies suggest that similar proportions of patients respond favourably to seasonal influenza vaccination regardless of methotrexate or tofacitinib exposure, and that vaccination could be undertaken yearly (as recommended) regardless of such therapy.

The impact of biologic and non-biologic DMARDs upon influenza and PPSV-23 immune responses in patients with RA is an important topic, and many studies have been reported. For non-biologic DMARDs, the effect of methotrexate on immunisation responses is best studied, and for PPSV-23, its negative effects are well documented.^25^ Data are varied for anti-tumour necrosis factor (anti-TNF) therapy and PPSV-23 immunogenicity: some studies have reported a decrease, whereas others suggest little or no effect.^26^ ^27^

A recent study evaluating tocilizumab in patients with RA demonstrated no effect on PPSV-23 response.^28^ Rituximab, however, severely diminishes humoral immune responses to PPSV-23, likely due to the drug's effects on B lymphocytes and the humoral immune response.^26^ Lastly, most patients vaccinated with PPSV-23 following abatacept exposure mount protective responses; however, similar to our data with tofacitinib, abatacept has been associated with decreased serotype-specific GMFR.^27^

For influenza vaccine, similar studies with DMARDs have been conducted.^29^ ^30^ Most studies on methotrexate showed mild or few negative effects on influenza vaccine immunogenicity. Similarly, most studies with anti-TNF therapy suggest such therapy did not hinder response to influenza vaccine, and a recent study with tocilizumab also suggested no negative effects.^13^ ^31^ ^32^ This is contrary to literature published for rituximab, where treatment severely reduced humoral responses to the influenza vaccine.^26^ ^33–35^ Patients receiving abatacept had reduced responses to monovalent pandemic influenza vaccine (trivalent vaccine was not evaluated) compared with those receiving methotrexate.^36^ While influenza responses are one indicator of responses to protein antigens, in controlled studies that have evaluated responses to tetanus toxoid, methotrexate alone leads to considerable reduction in responses to this protein antigen, an effect that did not significantly differ in combination with the addition of either rituximab or tocilizumab.^26^ ^37^

The tofacitinib studies presented herein were not designed to investigate the mechanism of action by which tofacitinib or methotrexate modulates T-cell-dependent or independent humoral responses. While methotrexate is well documented to interfere with the T-cell-independent humoral response to PPSV-23, the mechanism by which this occurs is poorly described.^25^ The JAK-signal transducer and activator of transcription pathway is important for both innate and adaptive immunity,^38^ and tofacitinib modulates signalling of cytokines involved with humoral immunity. While a potential mechanism for diminished humoral vaccine responses is not clear, it is conceivable that interferon-driven T-cell and B-cell development is impaired after vaccination, or that tofacitinib might inhibit signalling by interleukin (IL)-6 and IL-21, which are known to stimulate B cells to differentiate into antibody-producing cells.^39^ Most recently, tofacitinib has been shown to reduce B cell activation and IgG production.^40^

Current literature suggests that rheumatologists do not routinely address the need for vaccination prior to starting therapy with biologic DMARDs, and for the most part, recommended vaccines are used infrequently among patients with RA.^41–43^ While our study addressed two of the most important recommended vaccines, it should be noted that a newly available protein-conjugate vaccine for pneumococcus is recommended for patients with RA in the USA and other regions.^44^ ^45^ The pneumococcal conjugate vaccine (PCV-13) provides protection against 13 serotypes, 12 of which are contained within the PPSV-23 vaccine. Early data suggest that a strategy of using PCV-13 prior to PPSV-23 might improve the immunogenicity of PPSV-23.^45^ ^46^ Such a strategy deserves study in patients with RA.

In summary, we conducted two studies to evaluate the optimal timing of PPSV-23 and influenza vaccination in relation to tofacitinib use. Among patients newly starting tofacitinib, our results highlight that tofacitinib can diminish PPSV-23 immunogenicity to a similar extent as methotrexate, particularly when these two DMARDs are used concomitantly, while influenza responses are affected minimally. Consequently, clinicians should consider offering PPSV-23 prior to starting either of these therapies. Once patients are already using tofacitinib, our results suggest that temporary discontinuation of tofacitinib has little effect upon responses to either vaccine, and that the majority of patients will mount satisfactory responses to either vaccine.
